# Cholelithiasis and the risk of intrahepatic cholangiocarcinoma: a meta-analysis of observational studies

**DOI:** 10.1186/s12885-015-1870-0

**Published:** 2015-11-02

**Authors:** Hao Cai, Wen-Tao Kong, Chao-Bo Chen, Guo-Ming Shi, Cheng Huang, Ying-Hao Shen, Hui-Chuan Sun

**Affiliations:** 1 Liver Cancer Institute and Zhongshan Hospital, Fudan University, Shanghai, 200032 China; 2 Department of Ultrasound, Zhongshan Hospital Fudan University, Shanghai, 200032 China; 3 Department of General Surgery, Wuxi Xishan People’s Hospital, Wuxi, Jiangsu Province 214011 China

**Keywords:** Intrahepatic cholangiocarcinoma, Cholelithiasis, Choledocholithiasis, Cholecystolithiasis, Risk factors, Meta-analysis

## Abstract

**Background:**

The etiological factor for intrahepatic cholangiocarcinoma (ICC) is not clear. Although it has been widely accepted that intrahepatic biliary tree stone is associated with increased risk of ICC, the role of extrahepatic biliary tree stone in the incidence of ICC is controversial. In the present study we aim to evaluate the association between pre-existing choledocholithiasis and cholecystolithiasis and the risk of ICC.

**Methods:**

PubMed, Embase, and Web of Science were searched to identify cohort and case–control studies on the association between choledocholithiasis or cholecystolithiasis and the risk of ICC. Studies that met the inclusion criteria were subjected to a meta-analysis performed with Stata statistical software. Either a fixed or random effect model was used, depending on the heterogeneity within the studies. Egger’s test was performed to assess publication bias.

**Results:**

Seven case–control studies met our inclusion criteria. Of the 123,771 participants, 4763 (3.85 %) were patients with ICC, and 119,008 were tumor-free controls. The presence of pre-existing bile duct stones (choledocholithiasis alone or choledocholithiasis accompanied by hepatolithiasis) was associated with the risk of ICC (odds ratio [OR] 17.64, 95 % confidence interval [CI] 11.14–27.95). Even the presence of choledocholithiasis alone (in the absence of hepatolithiasis) was associated with a high risk of ICC (OR 11.79, 95 % CI 4.17–33.35). Cholecystolithiasis may possibly contributed to the incidence of ICC (OR 2.00, 95 % CI 1.16–3.42), with large heterogeneity within studies (*I*^2^ = 78.5 %).

**Conclusions:**

Bile duct stones including choledocholithiasis are important risk factors for ICC. Careful surveillance of patients with extrahepatic biliary tree stone should be considered.

**Electronic supplementary material:**

The online version of this article (doi:10.1186/s12885-015-1870-0) contains supplementary material, which is available to authorized users.

## Background

Biliary tract neoplasms are classified as intrahepatic cholangiocarcinoma (ICC), perihilar cholangiocarcinoma, or extrahepatic cholangiocarcinoma depending on the tumor location within the biliary tree [1]. ICC, which is defined as being located proximally to the second-order bile ducts, accounts for 10 % of total biliary tract neoplasms [2]. ICC, the second most frequent liver neoplasm following hepatocellular carcinoma, is highly malignant and shows extremely poor prognosis [3]. The incidence of ICC is relatively low but increasing worldwide [4, 5]. The risk factors for ICC are complex. Hepatolithiasis (which, along with cholecystolithiasis and choledocholithiasis, is a common lithiasis arising from certain part of the biliary tree) is an established risk factor for ICC, probably via repeated mechanical injury and inflammation of the intrahepatic biliary tract epithelium [4]. However, few studies have investigated the correlation between ICC and pre-existing extrahepatic biliary tract stones (choledocholithiasis or cholecystolithiasis). There is no consensus on whether choledocholithiasis or cholecystolithiasis contribute to the development of ICC. In the present study, we systematically reviewed the literature on the correlation between ICC and pre-existing cholelithiasis and performed a meta-analysis of relevant cohort and case–control studies to assess the risk of ICC in patients with pre-existing choledocholithiasis and cholecystolithiasis.

## Methods

### Selection of studies

PubMed, Embase, and Web of Science were searched using the following key words: ‘Cholelithiasis’ or ‘Choledocholithiasis’ or ‘Cholecystolithiasis’; ‘Intrahepatic cholangiocarcinoma’ or ‘cholangiocarcinoma’ or ‘bile duct neoplasms’; and ‘Risk factors’ through December 2014. No limitations were set for the language or the year of publication. The reference lists of the retrieved articles were manually searched so that no possibly useful information was missed.

**Fig. 1:**
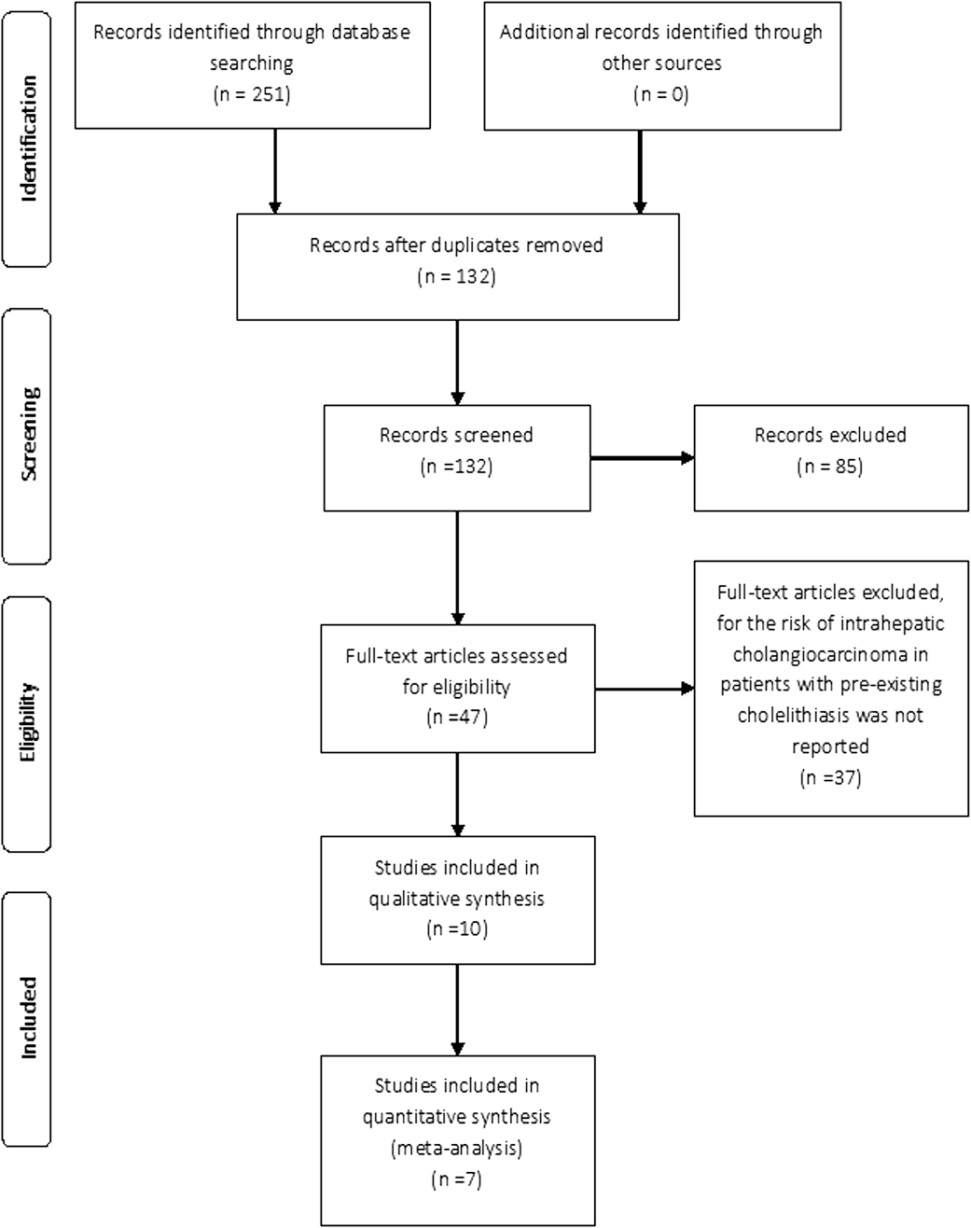
Flow diagram of study selection process

The inclusion criteria were as follows: (1) cohort or case–control studies of the correlation between ICC and pre-existing choledocholithiasis (with or without concurrent hepatolithiasis), or cholecystolithiasis independently; (2) studies in which the primary outcome was the occurrence of ICC; (3) studies in which the exposure of interest was the presence of either pre-existing choledocholithiasis (with or without concurrent hepatolithiasis) or cholecystolithiasis; and (4) studies in which estimates of relative risk (rate ratios, odds ratios [ORs], or standardized incidence ratios) with their 95 % confidence intervals (CIs) were available. Studies that enrolled patients with concurrent bile duct stones and cholecystolithiasis were excluded. The flow chart for selection of the studies is shown in Fig. 1.

### Data extraction

Two reviewers (WK and CC, both experts in the diagnosis and treatment of hepatobiliary diseases) independently extracted the data from the selected studies using a specially designed form. The following information was required for our study: name of first author, publication year, country or region, study design, number of cases (incidence of ICC in cohort studies), number of controls or cohort size, matched factors and confounders of each study. The validated Newcastle–Ottawa scale was used to assess the methodological quality of case–control and cohort studies [6]. Any discrepancy between the 2 reviewers in selecting publications and extracting data was resolved by discussion until a consensus was reached. When data on both bile duct stones (choledocholithiasis accompanied by hepatolithiasis) and choledocholithiasis alone were provided, only the data on choledocholithiasis were recorded.

### Statistical analysis

Meta-analysis was performed with Stata statistical software (ver. 12.0, Stata, College Station, TX, USA). Dichotomous variables were expressed as relative frequencies and were compared by means of the *χ*^2^ test. The Cohen’s Kappa was used to assess the inter-rater reliability for inclusion decision. Relative risks (ORs, risk ratios, and standard incidence rates) with their corresponding 95 % CIs were used to assess the association between the risk of the development of ICC and pre-existing biliary stone disease. *χ*^2^ and *I*^2^ tests were used to assess between-study heterogeneity; *I*^2^ values ≥ 25, 50 and 75 % were considered to indicate mild, moderate and high heterogeneity. Either a fixed or random effect model (Inverse Variance method) was used, depending on the between-study heterogeneity. Subgroup analysis was performed to identify confounding factors that could possibly contribute to between-study heterogeneity. Publication bias and other biases were assessed by means of Egger’s test. Trim and fill tests combined with conversion between different effect models were performed in sensitivity analysis. *P* ≤ 0.05 was considered to indicate statistical significance.

## Results

### Selection of studies

As shown in Fig. 1, the initial database search returned 251 studies. Among these, 7 case–control studies met our inclusion criteria and were included in our meta-analysis: 3 nationwide case–control studies and 4 hospital-based case–control studies from 3 cities in mainland China and 1 city in Turkey (see Additional file 1: Table S1); [7–13]. The methodological quality of 3 of the studies was rated as high (score ≥ 7); [8, 10, 12], and that of the 4 other studies was rated as moderate (4 ≤ score < 7); [7, 9, 11, 13]. A total of 123,771 participants were enrolled, including 4763 ICC patients and 119,008 tumor-free controls. There was a strong consistency between the 2 reviewer in study selection (Kappa = 0.86).

Nordenstedt et al. conducted a cohort study on the association between cholecystolithiasis and the risk of ICC in a Swedish population. However, patients who had both cholecystolithiasis and bile duct stones were not eliminated, so this study was excluded from our analysis [14]. Wu et al. reported a correlation between cholelithiasis and the risk of ICC, but their study was also excluded from our meta-analysis owing to the lack of detailed information on choledocholithiasis or cholecystolithiasis alone [15]. Welzel et al. (2007a) and Shaib et al. conducted similar case–control studies of cholelithiasis and the risk of ICC in the United States on the basis of the Surveillance, Epidemiology, and End Results database [7, 16]. However, the study of Welzel et al. [7] was published more recently, so the study of Shaib et al. was excluded.

### Data synthesis

#### Bile duct stone and the risk of ICC

**Fig. 2:**
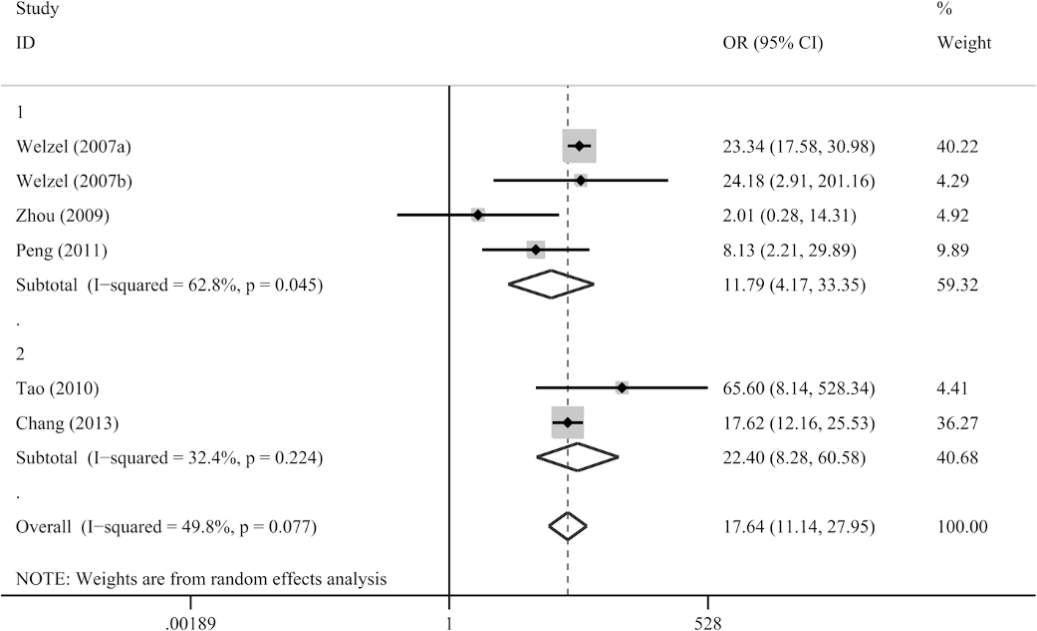
Forrest plot showing the correlation between bile duct stones and the risk of intrahepatic cholangiocarcinoma. Subgroup 1 included patients with only choledocholithiasis, whereas subgroup 2 included patients with both hepatolithiasis and choledocholithiasis

As shown in Fig. 2, 6 studies reported on the risk of ICC in patients with bile duct stones (choledocholithiasis with or without hepatolithiasis), with a pooled OR of 17.64 (95 % CI 11.14–27.95). There was no incidence of ICC in patients with pre-existing choledocholithiasis in the study of Ibrahim et al., which was therefore excluded [13]. A subgroup analysis including the 4 studies that reported on choledocholithiasis alone and the risk of ICC showed a pooled OR of 11.79 (95 % CI 4.17–33.35). There was mild heterogeneity within studies (*I*^2^ = 49.8 %, *p* = 0.077), and a random effect model was used. Subgroup analysis based on different regions, study designs and NOS scores, which are possible confounding factors was performed and shown in Table 1. The risk of ICC in patients with bile duct stones (choledocholithiasis with or without hepatolithiasis) remained statistically significant by different regions, study designs or NOS scores.

**Table 1:** Subgroup analysis according to region, study design and NOS score

			Heterogeneity	
Confounding factors	NO. studies	Odds ratio (95 % CI)	*I* ^*2*^	*p*
Region				
Eastern				
BDST	4	12.34 (4.54–33.57)	59.1 %	0.062
GBST	4	1.77 (0.88–3.58)	85.7 %	0.000
Western				
BDST	2	23.35 (17.63–30.92)	0.0 %	0.974
GBST	2	2.81 (1.03–7.72)	36.3 %	0.210
Study design				
Nationwide				
BDST	3	21.07 (16.85–26.36)	0.0 %	0.494
GBST	2	2.78 (2.37–3.26)	0.0 %	0.328
Hospital-based				
BDST	3	9.75 (1.73–54.84)	62.5 %	0.056
GBST	4	1.42 (0.63–3.19)	70 %	0.019
NOS score				
High				
BDST	3	18.49 (12.90–26.50)	0.0 %	0.463
GBST	3	2.58 (1.59–4.21)	53.2 %	0.118
Moderate				
BDST	3	9.53 (2.55–35.59)	75.2 %	0.018
GBST	3	1.57 (0.54–4.61)	79.4 %	0.008

*CI* confidence interval, *EHST* extrahepatic bile duct stone or choledocholithiasis, *GBST* gallbladder stone or cholecystolithiasis

#### Cholecystolithiasis and the risk of ICC

**Fig. 3:**
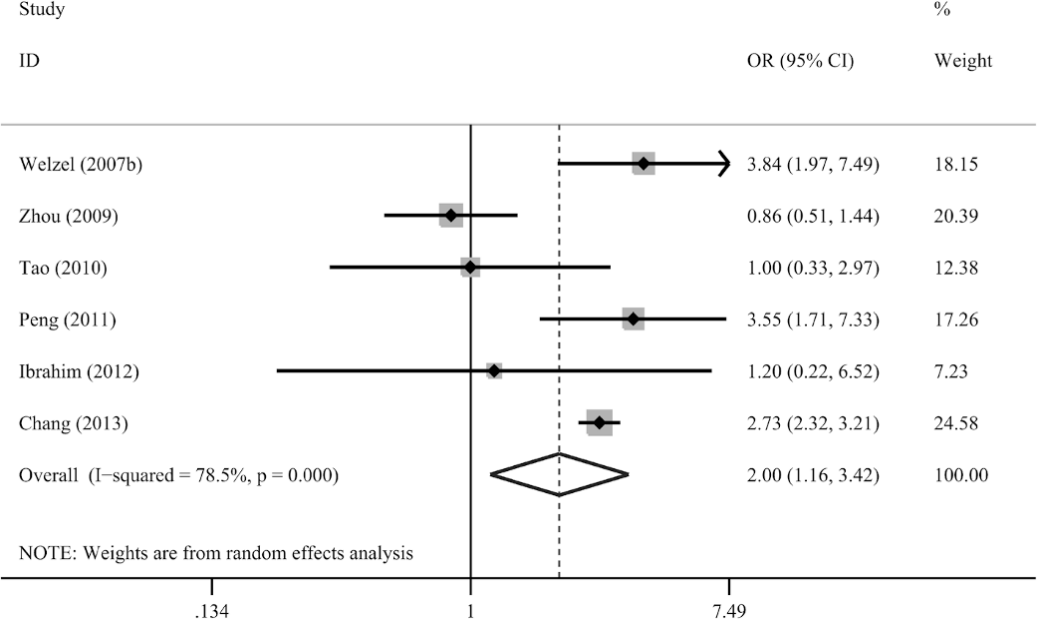
Forrest plot showing the correlation between cholecystolithiasis and the risk of intrahepatic cholangiocarcinoma

As shown in Fig. 3, 6 of the 7 studies reported information on cholecystolithiasis alone and the risk of ICC [8–13], with a pooled OR of 2.00 (95 % CI 1.16–3.42); the study of Welzel et al. [7] in a United States population did not provide detailed information on cholecystolithiasis separately, so this study was excluded from this meta-analysis. There was high heterogeneity within studies (*I*^2^ = 78.5 %, *p* = 0.000). Subgroup analysis based on different regions, study designs and NOS scores was performed and shown in Table 1. The outcome on the risk of ICC in patients with cholecystolithiasis was substantially altered and no statistically significant difference was observed with meta-analysis of studies from eastern countries, hospital-based studies and studies of lower NOS scores.

### Publication bias and sensitivity analysis

Egger’s test showed no evidence of publication bias for the meta-analysis of bile duct stones (*t* = −2.37, *p* = 0.077). However, biases existed in the meta-analysis of cholecystolithiasis (*t* = 2.81, *p* = 0.048), which could be due to high heterogeneity within studies. A sensitivity analysis was performed. Trim and fill analysis showed that the outcomes were not changed without trimming performed, and the outcomes still showed statistical significance when a fixed effect model was used.

## Discussion

Various established risk factors are associated with the development of ICC, including biliary parasitic infection, hepatolithiasis, bile duct cysts, primary sclerosing cholangitis, and exposure to certain toxins [4, 17]. In East Asian countries, hepatolithiasis and biliary parasitic infection are more common risk factors, whereas in Western countries, primary sclerosing cholangitis is the main risk factor for ICC [4]. Several systematic reviews and meta-analyses have suggested that there are correlations between ICC and pre-existing diabetes, obesity, and hepatic virus infections [18–21].

In the present study, we found that both choledocholithiasis and cholecystolithiasis were risk factors for the development of ICC, with the risk being higher for choledocholithiasis (OR 11.79, 95 % CI 4.17–33.35). There is a strong correlation between hepatolithiasis and ICC as confirmed by literature, which is in accordance with our findings [4]. Subgroup analysis showed that the ICC risk was lower for choledocholithiasis alone than for choledocholithiasis accompanied by hepatolithiasis. Even so, choledocholithiasis alone was still associated with a high risk of developing ICC. The mechanism by which choledocholithiasis might lead to the development of ICC remains unclear; cholestasis, changes in bile composition, and relevant metabolic syndromes may be involved. In addition, choledocholithiasis that drops down from the upstream intrahepatic biliary tract may result in chronic inflammation of the intrahepatic bile duct epithelium. The relationship between cholecystolithiasis and the risk of ICC is controversial; some studies show no correlation between ICC and pre-existing cholecystolithiasis [9, 10]. Our meta-analysis showed that cholecystolithiasis was associated with the risk of developing ICC, with significant between-study heterogeneity, which should be interpreted with caution. Choledocholithiasis is usually accompanied by various metabolic diseases such as diabetes and hyperlipidemia, which have been shown to be correlated with the development of ICC [18, 21–25].

Statistically significant heterogeneity existed within the studies included in the meta-analysis for cholecystolithiasis, which may be attributable to differences in regions (eastern versus western countries), study designs (nationwide versus hospital-based study), and Newcastle–Ottawa scale scores (high versus moderate quality), as shown in Table 1. Nationwide studies, studies of high quality or studies enrolling participants from western countries are more likely to produce stable outcomes with low heterogeneities. The outcome on the risk of ICC with pre-existing bile duct stones was not substantially altered, while it should be interpreted with caution for the correlation between ICC and pre-exsiting cholecystolithiasis. Smaller sample sizes in hospital-based studies may result in larger selection bias of cases and controls. Different study designs may lead to different Newcastle–Ottawa scale scores or affect the methodological quality of studies, which may account for biases in the confirmation of exposures and comparability between cases and controls. Besides, the role of cholelithiasis in the development of ICC may be diverse in different regions. In all but one of the included studies [7], age and sex were matched between cases and controls. It has been reported that old men may have a higher risk of developing ICC [4]. When we omitted the study of Welzel et al. [7] from the meta-analysis for choledocholithiasis, the outcome still remained statistically significant. Egger’s test showed no obvious publication bias, and sensitivity analysis showed that the outcome of the meta-analysis was stable.

Our meta-analysis does have some limitations. First, the evidence levels of the included case–control studies were relatively low, and there were no qualified cohort studies. Cholelithiasis is usually accompanied by other metabolic syndromes, which are also risk factors for the development of ICC [22–25]. To rule out the effects of these other factors, meta-analyses of qualified cohort studies will be essential. Second, the number of included studies was small. Our meta-analysis included patients from mainland China, Taiwan, the United States, Denmark and Turkey. Evidence has shown that the incidence of ICC varies geographically, with the highest incidence rate being in Thailand, which may be due to the high incidence of parasitic infections and hepatolithiasis there [26, 27]. In addition, the prevalence of hepatitis infection, which is also a risk factor for ICC, is higher in East Asian countries than in Western countries, which is consistent with the higher prevalence of ICC in East Asian countries [4, 19–21]. Also, the difference may be related to the genetic backgrounds of different races [28, 29]. In the future, a greater number of qualified studies from different regions and different ethic groups are needed to draw a more conclusive result. In addition, different pathological types of ICCs may go through different pathogenesis [30]. Also, the correlation between ICC and the duration, size, and number of stones needs further interpretation [31, 32].

As far as we know, this is the first systematic review and meta-analysis of studies evaluating the association between the risk of developing ICC and pre-existing choledocholithiasis and cholecystolithiasis, and our study may be of value for clinical practice. The prognosis of ICC is extremely poor, so early diagnosis and timely treatment of this highly malignant disease are important. Our evidence-based study showed that patients with a history of cholelithiasis, especially choledocholithiasis, are at high risk of developing ICC. Therefore, routine follow-up for these patients is critical for the early diagnosis of ICC. Early diagnosis and timely treatment can be expected to lead to better outcomes for ICC patients.

## Conclusions

Bile duct stones were found to be important risk factors for the development of ICC. Even in the absence of hepatolithiasis, choledocholithiasis was associated with a high risk of ICC.

## Additional file

Additional file 1: **Title of dataset: Data extracted from the studies included in the meta-analysis.** NOS Newcastle-Ottawa scale, CI confidence interval, CC case–control study, EHST extrahepatic bile duct stone or choledocholithiasis, GBST gallbladder stone or cholecystolithiasis, BDST bile duct stone, CLD chronic liver diseases, DM diabetes mellitus, ALD alcoholic liver disease, IBD inflammatory bowel disease, HBV hepatitis B virus. (DOC 62 kb)
