# Supplementary material for: Cholelithiasis and the risk of intrahepatic cholangiocarcinoma: a meta-analysis of observational studies
Source: BMC Cancer. 2015 Nov 2;15:831. doi: 10.1186/s12885-015-1870-0 (PMC4629290; doi:10.1186/s12885-015-1870-0)
Supplement: Additional file 1: — Title of dataset: Data extracted from the studies included in the meta-analysis. NOS Newcastle-Ottawa scale, CI confidence interval, CC case–control study, EHST extrahepatic bile duct stone or choledocholithiasis, GBST gallbladder stone or cholecystolithiasis, BDST bile duct stone, CLD chronic liver diseases, DM diabetes mellitus, ALD alcoholic liver disease, IBD inflammatory bowel disease, HBV hepatitis B virus. (DOC 62 kb) [file 12885_2015_1870_MOESM1_ESM.doc]

**Additional file 1: Table S1 - Data extracted from the studies included in the meta-analysis**

| First author | Publication year | Country or region | Study period | Study design | NOS score | No. cases | No. controls | Risk factor | Odds ratio (95% CI) | Matched factors | Confounders |
| --- | --- | --- | --- | --- | --- | --- | --- | --- | --- | --- | --- |
| Welzel et al. | 2007a | United States | 1993-1999 | Nationwide CC | 6 | 535 | 102,782 | EHST | 23.34 (17.58–30.98) | Year of search for risk factors | Age, sex, race, geographic location, other biliary tract conditions, CLD, DM, digestive disorders, smoking |
| Welzel et al. | 2007b | Denmark | 1978-1991 | Nationwide CC | 7 | 764 | 3,056 | EHST | 24.18 (2.91–201.16) | Age, sex, time of cancer diagnosis and control selection | ALD, Nonspecific cirrhosis, Cholangitis, IBD |
|  |  |  |  |  |  |  |  | GBST | 3.84 (1.97–7.49) |  |  |
| Zhou et al. | 2009 | Shanghai, China | 2003-2006 | Hospital-based CC | 5 | 317 | 634 | EHST | 2.01 (0.28–14.31) | Age, sex, nationality, residence | HBV infection, cirrhosis, Schistosomiasis |
|  |  |  |  |  |  |  |  | GBST | 0.86 (0.51–1.44) |  |  |
| Tao et al. | 2010 | Beijing, China | 1998-2008 | Hospital-based CC | 7 | 61 | 380 | BDST | 65.60 (8.14–528.34) | Age, sex, residence | HBV infection, History of cholecystectomy |
|  |  |  |  |  |  |  |  | GBST | 1.00(0.33–2.97) |  |  |
| Peng et al. | 2011 | Nanning, China | 2002-2009 | Hospital-based CC | 5 | 98 | 196 | EHST | 8.13 (2.21–29.89) | Age, sex, time of cancer diagnosis and control selection | HBV infection, cirrhosis, Liver fluke infestation |
|  |  |  |  |  |  |  |  | GBST | 3.55 (1.71–7.33) |  |  |
| Ibrahim et al. | 2012 | Ankara, Turkey | 2006-2010 | Hospital-based CC | 5 | 10 | 48 | EHST | - | Age, sex | Smoking |
|  |  |  |  |  |  |  |  | GBST | 1.20 (0.22-6.52) |  |  |
| Chang et al. | 2013 | Taiwan | 2004-2008 | Nationwide CC | 7 | 2,978 | 11,912 | BDST | 17.62 (12.16–25.53) | Age, sex, time of cancer diagnosis and control selection | Cholangitis, cholecystitis, choledochal cyst, CLD, DM, digestive diseases |
|  |  |  |  |  |  |  |  | GBST | 2.73 (2.32–3.21) |  |  |

NOS Newcastle-Ottawa scale, CI confidence interval, CC case–control study, EHST extrahepatic bile duct stone or choledocholithiasis, GBST gallbladder stone or cholecystolithiasis, BDST bile duct stone, CLD chronic liver diseases, DM diabetes mellitus, ALD alcoholic liver disease, IBD inflammatory bowel disease, HBV hepatitis B virus
